# Medical Service Quality, Efficiency and Cost Control Effectiveness of Upgraded Case Payment in Rural China: A Retrospective Study

**DOI:** 10.3390/ijerph15122839

**Published:** 2018-12-13

**Authors:** Ruibo He, Ting Ye, Jing Wang, Yan Zhang, Zhong Li, Yadong Niu, Liang Zhang

**Affiliations:** School of Medicine and Health Management, Huazhong University of Science and Technology, Wuhan 430030, China; heruibo27@163.com (R.H.); yeting868@gmail.com (T.Y.); jingwang@hust.edu.cn (J.W.); zhangyan1604@163.com (Y.Z.); lizhong@hust.edu.cn (Z.L.); nyadong@126.com (Y.N.)

**Keywords:** case payment, upgraded version, quality, efficiency, cost control, rural China

## Abstract

**Background:** As the principal means of reimbursing medical institutions, the effects of case payment still need to be evaluated due to special environments and short exploration periods, especially in rural China. **Methods:** Xi County was chosen as the intervention group, with 36,104, 48,316, and 59,087 inpatients from the years 2011 to 2013, respectively. Huaibin County acted as the control group, with 33,073, 48,122, and 51,325 inpatients, respectively, from the same period. The inpatients’ information was collected from local insurance agencies. After controlling for age, gender, institution level, season fixed effects, disease severity, and compensation type, the generalised additive models (GAMs) and difference-in-differences approach (DID) were used to measure the changing trends and policy net effects from two levels (the whole county level and each institution level) and three dimensions (cost, quality and efficiency). **Results:** At the whole-county level, the cost-related indicators of the intervention group showed downward trends compared to the control group. Total spending, reimbursement fee and out-of-pocket expense declined by ¥346.59 (*p* < 0.001), ¥105.39 (*p* < 0.001) and ¥241.2 (*p* < 0.001), respectively (the symbol ¥ represents Chinese yuan). Actual compensation ratio, length of stay, and readmission rates exhibited ascending trends, with increases of 7% (*p* < 0.001), 2.18 days (*p* < 0.001), and 1.5% (*p* < 0.001), respectively. The intervention group at county level hospital had greater length of stay reduction (¥792.97 *p* < 0.001) and readmission rate growth (3.3% *p* < 0.001) and lower reimbursement fee reduction (¥150.16 *p* < 0.001) and length of stay growth (1.24 days *p* < 0.001) than those at the township level. **Conclusions:** Upgraded case payment is more reasonable and suitable for rural areas than simple quota payment or cap payment. It has successfully curbed the growth of medical expenses, improved the efficiency of medical insurance fund utilisation, and alleviated patients’ economic burden of disease. However, no positive effects on service quality and efficiency were observed. The increase in readmission rate and potential hidden dangers for primary health care institutions should be given attention.

## 1. Introduction

With people’s increasing awareness and need for accessible medical services, the prevalence of chronic diseases and the increasing trend of aging, among other factors, rising health expenditures have become a financial challenge for many countries and have increased patients’ economic burden of disease. In 2016, about one-third of OECD (Organisation for Economic Cooperation and Development) countries’ health expenditure accounted for 11% of their GDP (Gross Domestic Product). In the United States, the proportion of health expenditure is as high as 17.2% and may reach 19.6% in 2024 [1]. Although the figure in China is relatively low (5.5%) [2], the government’s health spending has risen from ¥177.9 billion to ¥1.4 trillion (the symbol ¥ represents Chinese yuan), and per capita health expenses have increased from ¥748.8 to ¥3351.7 in the last 10 years [3]. Solving the problem of “kan bing nan, kan bing gui” (“getting medical care is difficult and expensive” [4]) has become an important issue in the new round of medical reform in China.

Given the information asymmetry and the absence of a cost control mechanism in the medical service system, the fee for service payment is more inclined to encourage supplier-induced demand, which is widely considered as the main reason for the inexorable growth of health expenditure [5]. Therefore, the shift from post-payment to pre-payment has become a critical strategy to control costs and improve the service efficiency of medical institutions. Case payment has gradually become the principal means of reimbursement in many developed countries since the introduction of diagnosis-related groups (DRGs) for U.S. Medicare patients in 1983 [6]. The core concept of case payment is to pre-set a fixed reimbursement amount to medical institutions. No compensation or partial compensation fee is given to suppliers if the actual expenses exceed this set quota [7], thereby constraining physicians’ behaviour and prompting them to provide effective services within a reasonable range [8].

During implementation, case payment can be roughly divided into two versions. The elementary version sums the total medical expenses that cover the entire treatment process of a single disease [9], such as quota payment or cap payment [10,11]. This simple design has a low inclusion rate and high operating and managing costs because of the complexity of the disease. The sophisticated version calculates expenses according to patient groups who are clinically similar and have the same hospital resource utilisation by age, gender and diagnosis complications [12]. Examples of this version are DRGs in the United States and many European countries [13] and diagnosis procedure combination in Japan [14]. This design may induce physicians to treat patients whose costs are lower than the associated reimbursement, up-coding and shift the costs to other agencies. Both versions offer no incentives for the provider to assure service quality [6].

With universal health insurance coverage (which accounted for 96.3% of the population in 2014 [15]) and improvement of the compensation ratio, reimbursement fees have become the main source of revenue for medical institutions in China, which makes the payment method become the main strategy for regulating the behaviour of providers [16], in order to promote reasonable treatment, examinations, and drug utilization, and reduce patient burden of medical expenses. The Chinese government first issued a document of case payment in 2004, which required regions to implement pilot work [17]; the elementary version is mainly used in such implementations. After more than 10 years of exploration, the Chinese government began to promote case payment throughout the country in 2017 [18,19]. Existing studies have inferred that case payment is currently more ethically acceptable than fees for services in the context of China [20]. However, given that information systems and providers’ coding capability are lagging, the sophisticated version cannot be implemented in rural areas at present [21].

Previous studies on the policy effects of case payment in rural China obtained contradictory conclusions on cost control because of the limitations of the elementary version [22,23]. Moreover, the effects on service quality and efficiency have not received equal attention, and the chosen method and indicators are not well justified; thus, any related findings are not persuasive [24]. Therefore, the present study selected a representative practice of the upgraded case payment model in Xi County [25,26]. This model can be regarded as a preliminary version of DRGs because it combines the clinical pathway and the severity of the disease. This study also evaluated its impacts on costs, quality and efficiency comprehensively. We presume that our study provides a reference value for case payment policy evaluation and areas where DRGs are not available.

## 2. Methods

### 2.1. Study Setting and Intervention Assignment

Henan Province is a highly populous province and has the largest agricultural area in China [27]. Its resident population is 95.324 million, of which the rural population is 49.92 million or 51.5%. Its per capita GDP reached ¥42,363, ranking 20th among China’s 31 provinces and municipalities in 2016. Xi County is part of Xinyang City, located 79 km from the downtown area, in southern Henan Province. Like other regions, the county has also been plagued by the rapid growth of medical expenditure. Hospitalisation expenses increased by 14.5% every year from 2007 to 2010, according to a local health statistical report [28]. After summarising the experiences and shortcomings of the simple cap payment (low inclusion rate, high mutation rate, decline in medical quality) and with support from the World Bank’s Rural Health Project [29], the local government initiated an upgraded case payment reform to alleviate the pressure of insurance funds in the latter half of 2011.

Even in situations where patients have the same disease, the costs may be different because of diverse conditions. Therefore, the local insurance department divided diseases into three categories according to their condition: simple (without complications, requiring conventional therapy only), complex (with complications that need to be treated), and extremely complex (the worst condition, requiring changes in diagnosis and therapy). Consequently, medical institutions combined the current service capability and treatment norm to perform “localisation” adjustment on the basis of the national clinical pathway. The final A/B/C path (corresponding to simple, complex, and extremely complex, respectively) was developed upon the approval of health administrative and insurance departments. The proportion of the three paths was set to 7:2:1 according to “the law of large numbers” [30] of disease incidence, and the C-path was not implemented at the township level.

To safeguard the effectiveness of the cost control measure, medical institutions and insurance department negotiated expenses based on the localised pathway. A certain profit margin was set to ensure the successful implementation of payment reform (the balance rate of township health centre was controlled at 15–20%, and the balance rate of the county hospital was controlled within 15%). Ultimately, the total spending, reimbursement fees and out-of-pocket expenses were determined for each path of the disease.

### 2.2. Study Design and Variables 

**Figure 1:**
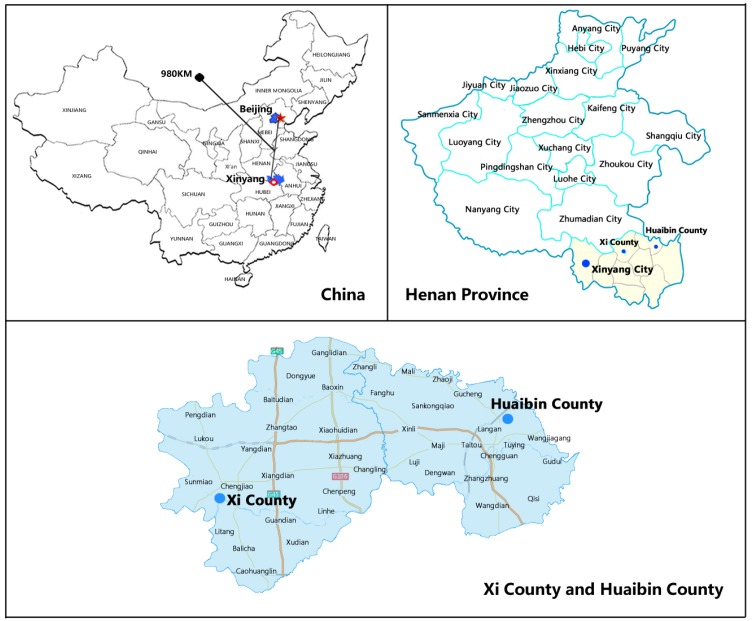
Locations of the Xi and Huaibin Counties in China and the Henan Province.

The reform was implemented throughout Xi County in January 2012 (the exploratory stage took place in the second half of 2011, and the pathway inclusion rate was less than 5%). Therefore, we used 2011 as the baseline period after excluding the few inpatients who were compensated through the upgraded model. To increase the reliability of the results, Huaibin County was chosen as the control group for three reasons. Firstly, its geographic proximity (see Figure 1) and population are similar to those of Xi County (Huaibin County was selected as the control group in previous studies on Xi County’s health care delivery system) [27]. Secondly, the two counties have similar health resources, service capability, and patients’ service utilisation habits (Table 1). Previous studies found that counties that did not implement payment reform had similar changes in their medical service-related indicators, which met the common trend assumption [16]. Thirdly, payment reform in Huaibin County is lacking (Huaibin began its global budget reform in 2014. Therefore, the time interval for this study is 2011 to 2013. Previously, inpatient service was still compensated by fee for service payment).

We intend to assess the effects from two levels (effects on the whole county and effects on different levels of medical institutions) and three dimensions (cost, quality and efficiency):

Although the county hospital and the township health centre can provide inpatient services, each of them has an explicit and relatively distinguished functional orientation. Township health centres play a pivotal role between village clinics and county hospitals and are responsible for primary care service of common diseases. County hospitals are the leaders of the three-tier health care delivery system in rural China and are committed to curative care for more complex diseases [31]. However, a trend exists in which patients seek higher-level medical services than what they actually need, which is one expression of irrational service utilisation [32,33,34]. The providers’ characteristics and behaviours will undoubtedly affect patients’ choice and health care outcomes [35]. Therefore, aside from evaluating the impact of reform on the overall county level, we also analysed medical institutions’ responses at the county and township levels.

The original purpose of case payment is to control costs, but its impact on efficiency and quality determines the achievement of its social goal. In the cost dimension, to make the result more specific, we analysed the effects on reimbursement fees, out-of-pocket expenses, and compensation ratio aside from total spending. The changes in length of stay before and after the intervention was compared to verify their effects on efficiency. The indicator of readmission within 30 days was determined to examine the effects on quality. All outcomes were calculated for every individual per inpatient visit [36]. The covariables contained individual traits (age, gender), level of medical institution, and disease severity (total spending was controlled to adjust the captured severity of illness. When the outcome variable was total spending, length of stay was controlled [36]), compensation type (whether or not patients were compensated by the upgraded version), year and season fixed effects. Data were obtained from the local health insurance agency and anonymised and de-identified before analysis.

### 2.3. Statistical Analyses

The difference-in-differences model (DID) is a scientific tool that is commonly used to evaluate intervention effects [37]. However, it can only show the final values and cannot reflect the changing trends of outcome variables during the intervention period. Therefore, we applied smoothing splines generated in generalised additive models (GAMs) [38] to indicate the effects between process and result.

GAMs, as semi-parametric extensions of generalised linear models, are applied to establish a relationship between the mean of the response variable and a “smoothed” function of the explanatory variables. Moreover, GAMs can deal with nonlinear and nonmonotonic relationships between the response and the set of explanatory variables with a link function [39]. Few studies have used GAMs in health policy evaluation and proved their validation [40,41]. In this part of the research, the “year” is the independent variable. After controlling the covariables, we constructed a smooth curve to observe the annual variation trends of the outcome variables of the intervention group and the control group at the whole county, county hospital, and township health centre levels.

To verify the trends reflected by the splines generated in GAMs and obtain more precise results, we adopted a DID using multivariate linear regressions, adjusted for covariables and time effects. In this part, three independent variables served as policy intervention indicators (0 = Huiabin County; 1 = Xi County). Given the hysteresis effects, a post-policy indicator was assigned as 0 for the pre-intervention period (between 1 January 2011 and 31 December 2011) and 1 for the post-intervention period (between 1 January 2013 and 31 December 2013), and their interaction term. The price index of rural health care was used to calibrate all spending figures according to 2013 Chinese yuan values [42]. The software packages R (http://www.R-project.org, The R Foundation, Mountain Ave, Murry Hill, NJ, USA) was used for all analyses. Results are reported with two-tailed p-values.

### 2.4. Ethics Statement

The research methods and investigation tools in this study were approved by the Ethics Committee of Tongji Medical College, Huazhong University of Science and Technology (IORG No: IORG0003571).

## 3. Results

### 3.1. Study Sample Characteristics

For consistency with DID analysis, Table 2 displays the information of the two groups in 2011 and 2013. The number of inpatients (male and female) in the intervention group was higher than that in the control group. The number of male inpatients was lower than that of female patients in both counties before and after the intervention. The 35–64 year age group was the main component, and the group over 65 years old showed an escalating trend. At the baseline, the mean values of total spending, reimbursement fee and out-of-pocket expense in the intervention group were all higher than those of the control group; these figures became lower than those of the control group after the intervention. Compensation ratio, length of stay, and readmission rate presented opposite changes. In terms of inpatient distribution, the number of township health centre visits in both counties dropped to about 8%, which equalled the increase in visits to the county hospital.

### 3.2. Policy Effects Shown by Observing Smooth Curve Trend

**Figure 2:**
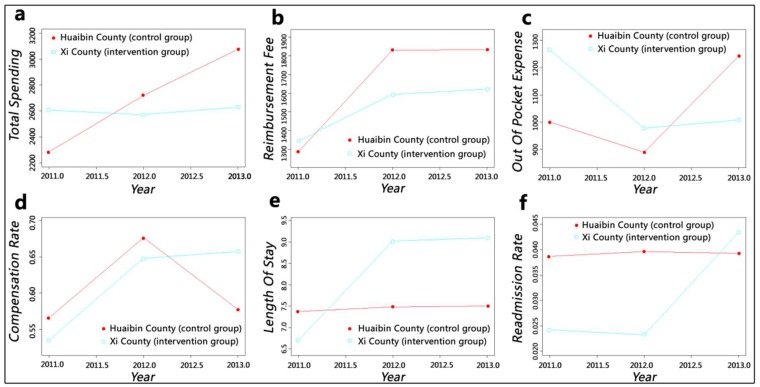
Smooth curves fitting for the whole county level: (**a**) adjusted mean of total spending; (**b**) adjusted mean of reimbursement fee; (**c**) adjusted mean of out-of-pocket expense; (**d**) adjusted mean of compensation ratio; (**e**) adjusted mean of length of stay; (**f**) adjusted mean of readmission rate.

At the whole-county level, the intervention of upgraded case payment resulted in evident changes among the independent variables of the intervention group (Figure 2). Unlike the continuous increase in the control group, the total spending of the intervention group showed a stable trend; the reimbursement fee of both groups increased, but the ascensional range of the intervention group was lower than that of the control group. The out-of-pocket expenses of the control group fluctuated, whereas those of the intervention group showed a considerable decline and then stabilised. The compensation ratio manifested an opposite trend. In terms of length of stay, the smoothing spline of the control group did not change distinctly, whereas that of the intervention group experienced a rapid growth in the first year and then stabilised. No major change in readmission rate was observed in the control group, whereas that of the intervention group was higher than that of the control group in 2013 after a considerable increase.

**Figure 3:**
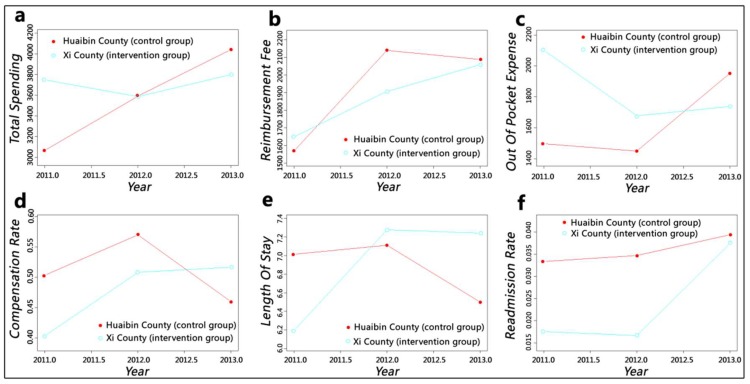
Smooth curves fitting for the county hospital level: (**a**) adjusted mean of total spending; (**b**) adjusted mean of reimbursement fee; (**c**) adjusted mean of out-of-pocket expense; (**d**) adjusted mean of compensation ratio; (**e**) adjusted mean of length of stay; (**f**) adjusted mean of readmission rate.

At the county hospital level, the changing trends of independent variables in intervention group were similar to those of the whole county level except for reimbursement fee, which grew consecutively and rapidly over the analysis period (Figure 3). The length of stay of the control group decreased markedly after a slight rise. At the baseline, the total spending, reimbursement fee and out-of-pocket expense values of the intervention group were higher than those of the control group but became lower than those of the control group after two years of policy implementation; the values of compensation ratio and length of stay showed opposite changes. The overall trends of readmission rate in both counties increased; although the readmission rate of the intervention group was visibly lower than that of the control group initially and showed a slight decline, it became close to that of the control group after a considerable growth.

**Figure 4:**
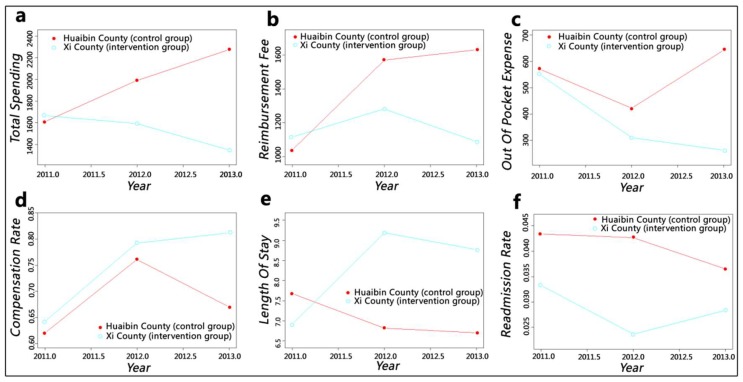
Smooth curves fitting for the township health centre level: (**a**) adjusted mean of total spending; (**b**) adjusted mean of reimbursement fee; (**c**) adjusted mean of out-of-pocket expense; (**d**) adjusted mean of compensation ratio; (**e**) adjusted mean of length of stay; (**f**) adjusted mean of readmission rate.

At the township health centre level, the cost-related indicators in intervention group all showed a decline trend (Figure 4). Total spending and out-of-pocket expense decreased steadily, and although reimbursement fee fluctuated, its reduction was greater than its growth. The changes of these trends in the control group were similar to that of the whole county and county hospital levels. The policy caused the compensation ratio of the intervention group to increase continually, and the length of stay declined after a considerable growth. Unlike the change of readmission rate of the control group, that of the intervention group rebounded after a decline.

### 3.3. Policy Effects Observed from Difference-In-Differences Approach (DID) Model Outcomes

The estimation at the whole-county level showed the effectiveness of cost control (Table 3): the total spending per capita decreased by ¥346.59 (*p* < 0.001), which amounted to a 21.63% decline. The contribution of out-of-pocket expense (reduction of ¥241.2 *p* < 0.001) was higher than that of reimbursement fee (¥105.39, *p* < 0.001). Therefore, compensation ratio increased by 7% (*p* < 0.001). However, the ungraded case payment reform led to an increase in length of stay and readmission rate by 2.18 days (*p* < 0.001) and 1.5% (*p* < 0.001), respectively.

The impact of cost control on total spending at the county hospital level was more apparent, with a reduction of ¥943.14 or a 57.88% decline. The reduction of reimbursement fee was slightly higher than that of the whole-county level, which was ¥150.16 (*p* < 0.001); the out-of-pocket expense faced the largest decline of ¥792.97 (*p* < 0.001), which was much greater than that of the whole county and township health centre levels. Compensation ratio increased the most with 14.4% (*p* < 0.001). The length of stay and readmission rate at the county hospital level also increased by 1.24 days (*p* < 0.001) and 3.3% (*p* < 0.001), respectively.

Total spending and reimbursement fee had the biggest drop of ¥1220.55 (*p* < 0.001) and ¥818.22 (*p* < 0.001) at the township health centre level. The change in out-of-pocket expense was significant but lower than that of the county hospital level, with a decline of ¥402.34 (*p* < 0.001). Compensation ratio also increased by 13.7% (*p* < 0.001). Length of stay had the largest increase of 4.45 days (*p* < 0.001), and readmission rate showed an insignificant reduction of 0.5% (*p =* 0.185). This result may be due to the fact that the increase of readmission rate at the whole-county level was caused by county hospitals.

## 4. Discussion

GAMs can intuitively describe the changing trends of the outcome variables during the intervention period, and the DID model can accurately reflect the changing values of the outcome variables. Therefore, we believe that the combination of these two methods can be used to evaluate the effects of a policy comprehensively and rationally.

If we merely focus on the cost control dimension, then our analysis shows encouraging results: the total spending at the whole county and county hospital levels showed a fluctuating but not rising trend and an obvious decline at the township hospital level (from smooth curves); the reduction degrees were 21.63%, 57.88%, and 108.53% at the whole county, county hospital, and township health centre levels, respectively (from DID estimation). However, our findings were inconsistent with two previous studies that also focused on Xi County payment reform; they believed that the reform failed to curb the cost growth, and total spending per capita continued to increase [21,28]. Three reasons were given for this conflict: Firstly, their research chose only one disease (caesarean section or chronic obstructive pulmonary disease), which cannot reflect and represent the impacts on the entire county. Secondly, they merely analysed the changes of intervention objects before and after the reform, and they lacked a suitable control group and did not control the time effects. Thus, the conclusions were not convincing. Thirdly, to successfully implement the reform at the starting period, some diseases’ profit margins were not reduced. However, the reform indeed cut off the direct link between cost and revenue.

With the enhancement of insurance funding, the annual premium per enrolee for the New Cooperative Medical Scheme was raised from ¥30 in 2003 to ¥570 in 2014. We can speculate that reimbursement fee should show an upward trend but still cannot keep up with the rising speed of expenditures, which caused an imbalance in the funding pool [43,44]. Figure 2 and Figure 3 show this trend; the growth range of the intervention group was lower than that of the control group. Moreover, reimbursement fee at the township level of the intervention group decreased after one year of policy implementation. Compensation ratio can reflect the efficiency of insurance fund utilisation. This value of the control group fluctuated to keep the funds balanced; although intervention group slowed down the increasing speed after a sharp growth at first year, it still kept rising. The compensation ratio of the whole county level increased by 7%, and the county and township levels increased by more than 10%. Therefore, the reform has effectively alleviated the pressure on medical insurance funds and improved the efficiency of its utilisation considerably. However, insurance funds flowed heavily into county hospitals, as indicated by a comparison of the findings at the county and township levels. This phenomenon could further widen the gap in service capacity between counties and towns and pose challenges to the development of township health centres.

The value of out-of-pocket expense can indicate patients’ economic burden of disease and shows an increasing trend with the rapid growth of health expenditures. Despite fluctuations, the out-of-pocket expense of the control group increased continually, whereas the out-of-pocket expense at the whole-county and county hospital levels of the intervention group showed a considerable downward trend even when it increased slightly in the second year. The out-of-pocket expense at the township health centre level declined steadily. The DID results were consistent with the GAMs: The county hospital had the largest reduction of ¥792.97, and the township health centre also dropped by ¥402.34. The reform has effectively alleviated inpatients’ economic burden in Xi County. However, this situation may lead to a hidden danger: aggravation of the previously mentioned issue of irrational medical service utilisation, which showed that patients tend to seek higher-level medical institution’s services instead of what they actually need. County hospitals have solved numerous diseases under township health centres’ service capacity [45], thereby increasing their bed occupancy rate to greater than 100% [46]. The reform has made the absolute value of the decline of the out-of-pocket expense in county hospitals much larger than that of the township health centre, which would cause patients to perceive that “hospitalisation services are much cheaper in county hospitals” and could simulate them to rely more on county hospital services.

Whether in rural or urban areas, medical institutions, especially high-level hospitals, are trying to shorten length of stay as much as possible and increase bed turnover to gain more benefits. The analysis results for the control group confirm this phenomenon. However, after the implementation of the upgraded case payment reform, the length of stay of the intervention group increased by 2.18 days, and the growth at the township level was higher than that at the county level. This phenomenon may be caused by the implementation of clinical pathways: physicians cannot decide patients’ discharge according to their clinical experience anymore. Rather, they must provide standard services every day based on the clinical path regulations, and they must consent to discharge only when the patients’ clinical condition meets the discharging indications within the specified time. Doing otherwise will be considered a violation, thereby affecting the compensation amount they receive.

Although the length of stay of the intervention group manifested a rising trend, we cannot conclude that the service efficiency was reduced, which should combine the changes of quality. The county-level readmission rate of the control group showed no obvious change, whereas that of the intervention group increased significantly. This result may be caused by decomposing hospitalisation, which is a disguised trick of medical institutions who aimed to obtain more insurance compensation [16]. Therefore, we can presume that the reform had not improved service quality and played no positive impact on service efficiency in the county. A similar situation was observed at the county hospital, and the growth range of readmission rate was greater. This issue needs to be taken seriously by insurance agencies, and the occurrence of readmissions should be reviewed strictly. This trend of the intervention group at the township level fluctuated and finally reached an insignificant decrease. We believed that the reform (mainly clinical pathway) had improved the service quality of township health centres and the growth of length of stay could not be simply ascribed to decreased efficiency. However, due to the previously mentioned issues which arise from changes in reimbursement fee and length of stay, the prospects of township health centres are not optimistic.

## 5. Limitations

Our study has several limitations. Firstly, we selected only one county as the intervention group and one county as the control group; both counties are located in one province. Thus, our results may not be generalisable to other counties in rural China. However, the reform in Xi County is representative across the country, and many counties have begun to follow suit. In the context of national promotion of case payment, our research offers reference value for other counties. Secondly, our analysis period lasted from 2011 to 2013 only because of the payment reform in the control group. Finding a county that maintains a unified payment method for a long time is difficult because the vast majority of regions in China are exploring payment reform. Meanwhile, study results confirmed the effectiveness of cost control of the upgraded case payment model and discovered some potentially hidden dangers that will guide future reform. Thirdly, although the reform is aimed at inpatient services, the study can perform a more comprehensive evaluation if it determines whether the reform leads to service transfer to outpatient, which is a subject of concern for future studies.

## 6. Conclusions

In general, the upgraded case payment reform successfully curbs the growth of medical expenses, improves the efficiency of medical insurance fund utilisation and alleviates patients’ economic burden of disease. Considering the complexity of the disease makes the payment design reasonable, and applying the clinical pathway improves the accuracy of cost calculation and can regulate and guide medical services. This payment reform provides a good inspiration for regions where DRGs are unavailable, especially in rural areas. A detail that should be noted is that the reform did not play a positive role in service quality and efficiency at the whole-county level. Moreover, the increase in readmission rate is an urgent problem that needs to be solved by health administration and medical insurance departments. In addition, the reform has resulted in insurance expenses being directed to county hospitals and may cause patients to opt for treatments at county hospitals. These phenomena go against the long-term development of primary healthcare institutions. Future payment reform should pay more attention to the health service delivery system, particularly with respect to cost, quality, and efficiency.

## Figures and Tables

**Table 1:** Socioeconomic Indicators of Xi and Huaibin Counties in 2015.

Indicators	Xi County	Huaibin County
Location	Contiguous to Huaibin	Contiguous to Xi
Administrative districts	21	19
Urbanization rate (%)	35.7	36.2
Permanent resident population (thousand)	833.5	565.7
Per capita annual income (yuan)	13,235	12,946
Number of county-level hospitals	3	3
Number of township hospitals	20	19
Number of hospital beds (per thousand population)	2.05	2.38
Number of practitioners (per thousand population)	1.36	1.48
Number of nurses (per thousand population)	1.07	1.11

**Table 2:** Basic characteristics of inpatients in Xi and Huaibin Counties, 2011 and 2013.

	Baseline (2011)			Follow-Up (2013)		
	Xi (Intervention)	Huaibin (Control)	*p*	Xi (Intervention)	Huaibin (Control)	*p*
*N*	36,104	33,073		59,087	51,325	
Gender			<0.001			<0.001
Male	15,173 (42.03%)	14,270 (43.15%)		25,816 (43.69%)	21,577 (42.04%)	
Female	20,931 (57.97%)	18,803 (56.85%)		33,271 (56.31%)	29,748 (57.96%)	
Age group			<0.001			<0.001
0–17	4897 (13.56%)	2944 (8.90%)		6630 (11.22%)	3887 (7.57%)	
18–34	9973 (27.62%)	6605 (19.97%)		12,711 (21.51%)	11,388 (22.19%)	
35–64	13,477 (37.33%)	15,554 (47.03%)		23,820 (40.31%)	23,078 (44.96%)	
≥65	7757 (21.49%)	7970 (24.09%)		15,926 (26.95%)	12,972 (25.27%)	
Institution level			<0.001			<0.001
Township level	17,921 (49.64%)	19,179 (57.99%)		24,788 (41.95%)	25,212 (49.12%)	
County level	18,183 (50.36%)	13,894 (42.01%)		34,299 (58.05%)	26,113 (50.88%)	
Total spending (¥)	2451.26	2103.90	<0.001	2690.09	3066.93	<0.001
Reimbursement fee (¥)	1302.53	1264.99	<0.001	1841.45	1898.87	<0.001
Out-of-pocket expense (¥)	1148.73	838.91	<0.001	848.65	1168.06	<0.001
Compensation ratio (%)	54.27	59.17	<0.001	69.46	59.18	<0.001
Length of stay (days)	7.05	7.71	0.558	9.43	7.85	<0.001
Readmission			<0.001			<0.001
No	35,006 (96.96%)	31,492 (95.22%)		55,853 (94.53%)	48,804 (95.09%)	
Yes	1098 (3.04%)	1581 (4.78%)		3234 (5.47%)	2521 (4.91%)	

The symbol ¥ represents Chinese yuan.

**Table 3:** Results of difference-in-differences analysis on policy effects.

Outcome Variable	2011 (Baseline)			2013 (Follow-up)			Difference-in-Differences
	Intervention	Control	Difference	Intervention	Control	Difference	
Whole county							
Total spending	1602.12	1180.07	422.04 ***	2212.46	2137.01	75.45 ***	−346.59 ***
Reimbursement fee	615.03	513.20	101.83 ***	1137.61	1141.17	−3.56	−105.39 ***
Out of pocket expense	987.09	666.87	320.21 ***	1074.85	995.84	79.01 ***	−241.20 ***
Compensation rate	0.448	0.487	−0.039 ***	0.517	0.486	0.031 ***	0.070 ***
Length of stay	3.41	4.08	−0.67 ***	4.89	3.38	1.51 ***	2.18 ***
Readmission rate	0.013	0.028	−0.016 ***	0.029	0.030	−0.001	0.015 ***
County hospital							
Total spending	1629.48	938.26	691.22 ***	1666.02	1917.94	−251.92 ***	−943.14 ***
Reimbursement fee	440.97	361.51	79.46 ***	815.77	886.47	−70.70 ***	−150.16 ***
Out-of-pocket expense	1188.51	576.75	611.75 ***	850.25	1031.47	−181.22 ***	−792.97 ***
Compensation rate	0.397	0.497	−0.100 ***	0.498	0.454	0.044 ***	0.144 ***
Length of stay	1.52	2.30	−0.78 ***	2.30	1.84	0.46 ***	1.24 ***
Readmission rate	0.005	0.025	−0.020 ***	0.046	0.034	0.013 ***	0.033 ***
Township health centre							
Total spending	1124.59	1073.83	50.76 ***	566.08	1735.87	−1169.79 ***	−1220.55 ***
Reimbursement fee	605.99	534.28	71.71 ***	379.19	1125.70	−746.51 ***	−818.22 ***
Out-of-pocket expense	518.60	539.54	−20.94 ***	186.89	610.17	−423.28 ***	−402.34 ***
Compensation rate	0.558	0.536	0.022 ***	0.745	0.587	0.159 ***	0.137 ***
Length of stay	3.17	3.87	−0.70 ***	6.66	2.91	3.75 ***	4.45 ***
Readmission rate	0.019	0.030	−0.011 ***	0.008	0.027	−0.016 ***	−0.005

Adjusted results are from a difference-in-differences model controlling for all covariates. *** *p* < 0.001.
